# Purtscher’s and Purtscher-like retinopathy etiology, features, management, and outcomes: A summative systematic review of 168 cases

**DOI:** 10.1371/journal.pone.0306473

**Published:** 2024-09-06

**Authors:** Hashem Abu Serhan, Mohammad T. Abuawwad, Mohammad J. J. Taha, Amr K. Hassan, Luai Abu-Ismail, Mohammad Delsoz, Hamzeh M. Alrawashdeh, Hamad A. Alkorbi, Obadah Moushmoush, Ayman G. Elnahry

**Affiliations:** 1 Department of Ophthalmology, Hamad Medical Corporation, Doha, Qatar; 2 Kasr Alainy Faculty of Medicine, Cairo University, Cairo, Egypt; 3 Gavin Herbert Eye Institute, University of California, Irvine, Irvine, California, United States of America; 4 Department of Ophthalmology, Islamic Hospital, Amman, Jordan; 5 Department of Ophthalmology, University of Tennessee Health Science Center, Memphis, TN, United States of America; 6 Department of Ophthalmology, National University Hospital, Singapore, Singapore; 7 College of Medicine, Qatar University, Doha, Qatar; 8 Department of Ophthalmology, MedStar Georgetown University Hospital, Washington, D.C., United States of America; 9 Bascom Palmer Eye Institute, University of Miami, Miami, FL, United States of America; 10 Department of Ophthalmology, Faculty of Medicine, Cairo University, Cairo, Egypt; Mount Saint Peter Eye Clinic, University of Tuebingen, GERMANY

## Abstract

**Background:**

To describe Purtscher’s and Purtscher-like retinopathy clinical features, etiologies, management options, and visual outcomes.

**Methods:**

Our protocol was registered on PROSPERO [registration number: **CRD42023406843**]. Seven online databases were searched: PubMed, Scopus, Medline, ScienceDirect, CENTRAL, clinicaltrials.gov, and Google Scholar. Original articles were included if they reported at least one subject diagnosed with Purtscher’s or Purtscher-like retinopathy. The primary outcome is to describe the clinical features of Purtscher and Purtscher-like retinopathies, including etiologies, results of related investigations, management lines, and visual outcomes. All analyses were conducted with the use of Statistical Package for Social Sciences (SPSS) version 27 (IBM SPSS Corp, SPSS Statistics ver. 26, USA) and Cochrane’s RevMan software. The methodological quality of included studies was assessed using the NIH quality assessment tools.

**Results:**

A total of 114 articles were included, describing 168 cases of Purtscher’s and Purtscher-like retinopathy. Patients were evenly distributed between males (50.89%) and females (49.11%). Average age of patients was 34.62 years old. Trauma was the leading cause of retinopathy, being reported in 39.88% of our patients, followed by systemic lupus erythematosus (SLE) (13.1%) and acute pancreatitis (11.9%). Bilateral symptoms were reported in 57.7% of patients with centrally blurred vision being the most complained symptom (OS: 34.32% and OD: 18%). 75% of patients elicited bilateral retinal findings. Cotton-wool spots were of highest prevalence (58%). Purtscher flecken was seen in 53% of patients. Macular edema was seen in 13% of patients. Overall, patients had a favorable prognosis (53%).

**Conclusion:**

Purtscher’s and Purtscher-like retinopathies are rare sight-threatening retinopathies that develop most commonly following trauma or other systemic diseases as SLE and acute pancreatitis. Little data is available regarding these conditions, and available data is of low quality. Patients develop bilateral disease in approximately 50% of cases, and several retinal findings are observed, with no specific tendency. Most observed signs are cotton-wool spots in around 55% of patients and Purtscher flecken in 51% of patients. Patients spontaneously recovered, although data is not conclusive. No clear prognostic value of etiological factors is identified, and further research is required in this regard.

## Background

First described in 1910 by Otmar Purtscher, Purtscher’s retinopathy is a form of traumatic retinal angiopathy. It is brought about by occlusive microvasculopathy which may be associated with intraretinal hemorrhages, and often presents with sudden regression of visual acuity, bilaterally, within two days of trauma. A few potential pathological mechanisms resulting in Purtscher’s retinopathy were described in literature, all of which suggest that the most likely cause of the flecken is a precapillary arteriolar occlusion due to an embolus. Some these mechanisms include raised intracranial pressure and extravasation of lymph, raised intrathoracic pressure and venous dilatation, and vasculitis due to free fatty acids [1]. Retinas affected by Purtscher’s retinopathy often elicit Purtscher flecken (areas of retinal whitening), retinal hemorrhages, and cotton-wool spots [2–4]. Purtscher-like retinopathy is a similar retinopathy that is not related to traumatic origin but rather develops in the setting of pancreatitis, kidney disease, malignancy, hemolytic uremic syndrome (HUS), autoimmune diseases (e.g. Systemic lupus erythematosus (SLE)), and several other systemic and infectious illnesses including the novel COVID-19 [5]. The incidence of Purtscher-like retinopathy has been linked to multiorgan failure or advanced disease, leading to fatality [6].

Diagnosis of Purtscher-like retinopathy is often done clinically. A history of etiological factors in association with clinical signs can be sufficient to diagnose patients. Purtscher flecken seen on fundoscopy are considered pathognomic, although not seen in 50% of patients, however; other findings like cotton-wool spots and intraretinal hemorrhages can also aid in diagnosis [2, 5, 7]. The main hypothesis for the pathophysiology of Purtscher-like retinopathy is an occlusion of peripapillary terminal arterioles. This occlusion occurs due to a sequence of inflammation, leukoembolization, endothelial damage, activation of complement C5, and C5a predisposition of leukocytes (granulocytes) aggregation [1, 8]. Around 60% of cases of this condition are bilateral [6]. The incidence of Purtscher’s retinopathy is not clear, as some reports have estimated the annual incidence of Purtscher’s and Purtscher-like retinopathies to be approximately 0.24 individuals per million, while others assumed the incidence to be higher since this condition could often be asymptomatic [5].

Overall, cases of Purtscher’s and Purtscher-like retinopathy are managed conservatively, with marked visual improvement spontaneously occurring. Nevertheless, some literature suggests that patients benefit from intravenous methylprednisolone pulse, suggesting it helps restore microvasculature and inhibit granulocyte aggregation. Otherwise, symptomatic treatment has been used, such as anti-vascular endothelial growth factor for cases with macular edema, steroids, and immunosuppressive therapy for autoimmune disease-related cases [9–12]. In this systematic review of the literature, we attempt to explore clinical features, causes, cross-correlations, management and prognostic factors in Purtscher’s and Purtscher-like retinopathy.

## Methods

This systematic review was conducted as per the recommendation of the PRISMA checklist for systematic reviews and meta-analyses (S1 Checklist). Our protocol was registered on PROSPERO prospectively [registration number: **CRD42023406843**]. This study adhered to the tenets of the Declaration of Helsinki. Given that our study did not involve human subjects, an institutional review board (IRB) approval was not required. On June 14, 2023, seven online databases were searched: PubMed, Scopus, Medline, ScienceDirect, Cochrane Central Register of Controlled Trials (CENTRAL), clinicaltrials.gov, and Google Scholar.

The search query was built following the PICO framework: participants were patients diagnosed with Purtscher’s or Purtscher-like retinopathy, and no restrictions applied on the interventions, comparators, and describing clinical features, etiologies, management options, and visual outcomes. In addition, no restrictions were applied depending on language, publication date, or study design. The primary outcome is to describe the clinical features of Purtscher and Purtscher-like retinopathies, including etiologies, results of related investigations, management lines, and visual outcomes. Secondary outcome included provide prognostic factors for Purtscher or Purtscher-like retinopathies related to the underlying etiology.

The search including the following keywords ((Purtscher[tiab] OR Purtscher-like[tiab] OR Pseudo-Purtscher) AND retinopath*[tiab]. The search criteria were modified as per the searched database. In addition, an updated manual search was performed to avoid missing any potentially related studies.

### Eligibility criteria

Original research articles were included if they reported at least one subject diagnosed with Purtscher’s or Purtscher-like retinopathy. In the meantime, studies were excluded if they met at least one of the following criteria: (1) articles not reporting the target population, (2) non-original research (i.e., reviews, commentaries, guidelines, editorials, correspondence, and letters to editors), (3) unavailable full texts, (4) abstract-only papers with no published full texts, (5) duplicated studies, and (6) studies with unextractable data.

## Selection of studies

Retrieved articles from electronic databases were imported in Endnote Software for duplicate removal. Then, citations were exported an Excel Sheet for screening. Screening was performed on two steps: title/abstract and full text screening. Two sets of two reviewers each [LAI and MD; HAA and OM] carried out the screening process, where the titles, abstracts, and full texts of all records were screened simultaneously by two reviewers. Differences between reviewers were solved by a thorough discussion, and when necessary, the senior authors [HAS and AGE] were consulted to give a final decision in unsolved disputes.

### Data extraction

Two reviewers [HAS and AGE] developed the data extraction sheet with the use of Microsoft Excel. The extraction sheet included three parts: baseline characteristics, main outcomes, and secondary outcomes. The first part included references’ information (i.e., author name, year of publication, study design, and sample size [number of patients and eyes]) and patients’ characteristics (i.e., intervention and comparison groups, type of retinopathy, age, and gender). The second part included data related to Purtscher’s or Purtscher-like retinopathy, such as the etiology, symptoms, signs, and management lines. The third part was related to the quality assessment and risk of bias of the include articles. Two sets of two reviewers each [LAI and MD; HAA and OM] extracted relevant data from finally included articles. Finally, two senior authors checked the accuracy of extracted data before the analysis [HAS and AGE].

### Risk of bias assessment

The methodological quality of included studies was assessed using the National Institute of Health (NIH) quality assessment tools (https://www.nhlbi.nih.gov/health-topics/study-quality-assessment-tools) for each respective study design included. The NIH tool assesses the included of studies at the level of several domains: research question, study population, sample size justification, inclusion and exclusion criteria prespecified and applied uniformly, case and control definitions, random selection of study participants, exposure assessed prior to outcome measurement, exposure measures and assessment, blinding of exposure assessors and statistical analysis. Each of these domains is given a final decision of no information or good (>7), fair (4–7), or poor (<4). Finally, each study is given a final evaluation based on the evaluation reported in all domains. The quality assessment was performed by two sets of two reviewers, where each article was assessed by [LAI and MD; HAA and OM]. Any differences between reviewers were solved by discussion, and the senior authors [HAS and AGE] were consulted to give a final decision regarding any unsolved disputes.

### Data synthesis and analysis

All analyses were conducted per-protocol (**CRD42023406843**) according to assessed patients with the use of Statistical Package for Social Sciences (SPSS) version 27 (IBM SPSS Corp, SPSS Statistics ver. 26, USA) and Cochrane’s RevMan software. We performed descriptive analysis presenting the categorical variables as percentages and frequencies while numerical variables as a mean and standard. The significance of the data was assessed using a categorical Chi-square test. All statistical tests were conducted with a 95% confidence interval and a 5% error margin. A p-value of less than 0.05 was considered statistically significant.

## Results

### Characteristics of the included studies

**Fig 1:**
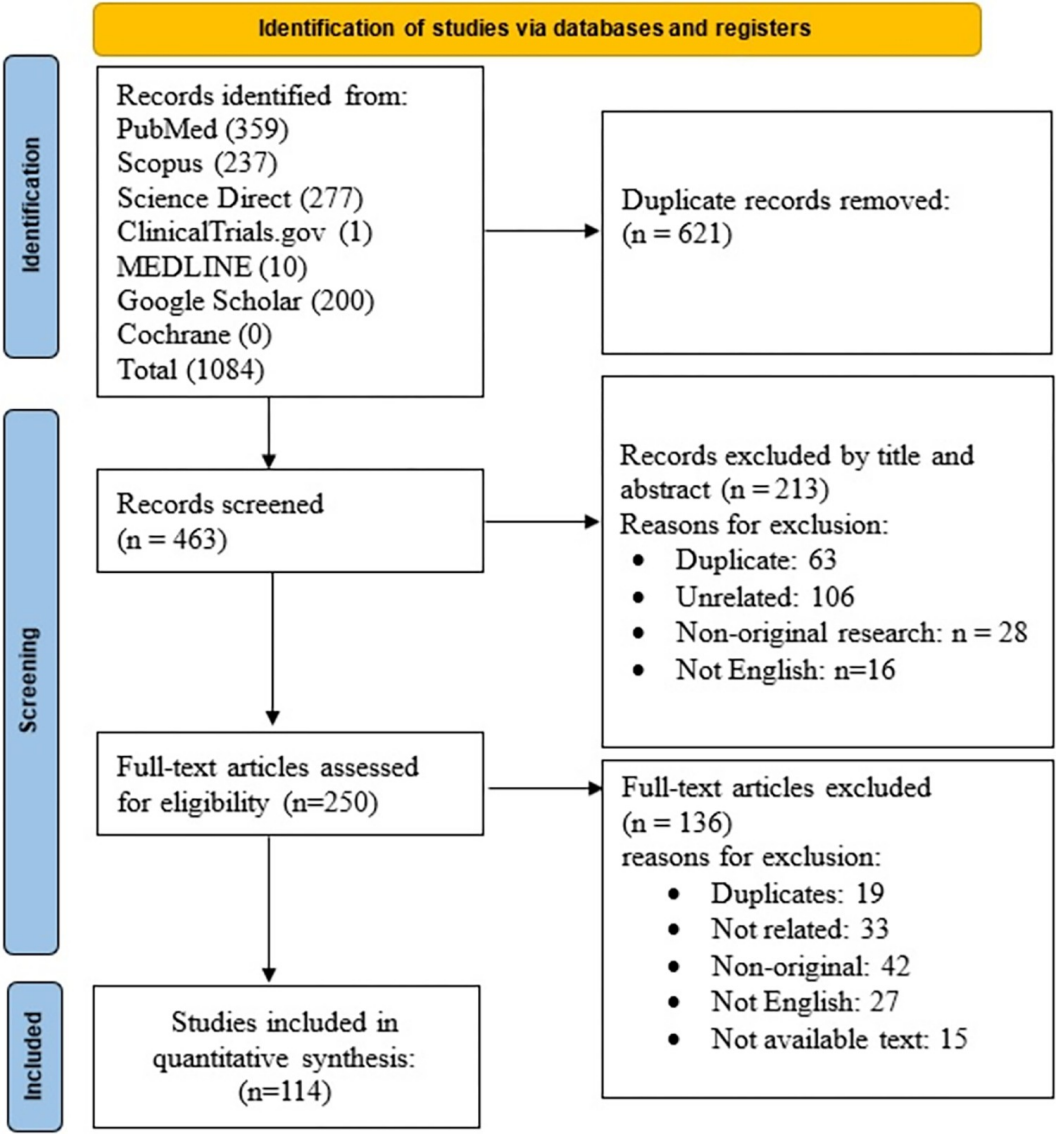
PRISMA chart describing the filtration process.

Our initial search concluded 1084 articles. Filtration was done according to the inclusion criteria, yielding 114 papers after the final stage. **Fig 1** describes the filtration process according to the PRISMA guidelines. Included articles described 168 cases of Purtscher’s or Purtscher-like retinopathy with ages ranging from 2–75 years, with an average of 34.62 years. Of patients, 49.11% were female, and cases were reported from countries all over the world. **Table 1** includes a summery for all included articles.

**Table 1:** Summary of included articles.

Study ID.	Country	Study Type	No. of patients	Age	Sex	Quality Assessment
Abo-Zed et al. [13]	USA	Case Report	1	34	Male	Poor
Akiyama et al. [14]	Japan	Case Report	1	29	Female	Fair
Alaei et al. [15]	Canada	Case Report	1	39	Female	Fair
Alahmadi et al. [16]	Saudi Arabia	Case Report	1	21	Female	Fair
Alsberge et al. [17]	USA	Case Report	1	46	Female	Fair
Alzahrani et al. [18]	Saudi Arabia	Case Report	1	37	Female	Poor
Arora et al. [19]	USA	Case Report	1	39	Female	Poor
Ayaki et al. [20]	Japan	Case Report	1	30	Female	Fair
Ayhan et al. [21]	Turkey	Case Report	1	36	Female	Fair
Baarsma & van Balen [22]	The Netherlands	Case Report	1	8	Male	Poor
Bader-Meunier et al. [23]	France	Case Report	1	7	Male	Fair
Battista et al. [24]	Italy	Case Report	1	59	Female	Poor
Bezzina et al. [25]	Malta	Case Report	1	30	Male	Fair
Bhan et al. [26]	UK	Case Series	3	49	2 Females, 1 Males	Poor
Blodi et al. [27]	USA	Case Series	4	23	4 Females	Good
Bui et al. [28]	USA	Case Report	1	32	Male	Fair
Burton et al. [29]	USA	Case Series	4	17.75	4 Males	Fair
Carvalho et al. [30]	Netherland	Case Report	1	20	Female	Fair
Castillo et al. [31]	USA	Case Report	1	20	Male	Fair
Castillo Jr et al. [32]	USA	Case Report	1	29	Female	Fair
Cernea et al. [33]	Romania	Case Report	1	44	Female	Fair
Chandra et al. [34]	India	Case Report	1	30	Male	Poor
Chebil et al. [35]	Tunisia	Case Report	1	55	Male	Fair
Cheng et al. [36]	China	Case Report	1	32	Female	Fair
Cheng et al. [37]	China	Case Report	1	38	Female	Fair
Coban-Karatas et al. [38]	Turkey	Case Report	1	31	Male	Fair
Cumurcu et al. [39]	Turkey	Case Report	1	11	Male	Fair
Devonport et al. [40]	UK	Case Report	1	21	Male	Fair
Durrani et al. [41]	United state	Case Report	1	44	Male	Fair
Dyrda et al. [42]	Spain	Case Report	1	50	Male	Fair
Elkaroui et al. [43]	France	Case Report	1	36	Female	Fair
Elwooda et al. [44]	United State	Case Report	1	75	Female	Poor
Engelmann et al. [45]	South Africa	Case Report	1	2	Male	Fair
Gahn et al. [46]	USA	Case Report	1	22	Male	Fair
Gange et al. [47]	USA	Case Report	1	38	Female	Fair
Gediz [48]	Turkey	Case Report	1	61	Male	Fair
Geraissate et al. [49]	Brazil	Case Report	1	2	Female	Fair
Giani et al. [50]	Italy	Case Report	1	31	Male	Fair
Gibson et al. [51]	UK	Case Report	1	38	Female	Fair
Grabe & Zacks [52]	United state	Case Report	1	53	Female	Fair
Gungel et al. [53]	Turkey	Case Report	1	51	Female	Fair
Gupta et al. [54]	United State	Case Report	1	25	Male	Fair
Haque et al. [55]	Bangladesh	Case Report	1	20	Male	Fair
Hashim et al. [56]	Qatar	Case Report	1	24	Male	Fair
Haslett et al. [57]	UK	Case Series	3	50.67	2 Males, 1 Females	Poor
Huang et al. [58]	United state	Case Report	1	20	Female	Fair
Huang et al. [59]	China	Case Report	1	18	Female	Fair
Hupy et al. [60]	USA	Case Report	1	25	Male	Fair
Hussain et al. [61]	United State	Case Report	1	24	Male	Poor
Inkeles et al. [62]	United State	Case Series	3	33.33	2 Males, 1 Females	Good
İpek et al. [63]	Turkey	Case Report	1	64	Male	Poor
Ishibashi et al. [64]	Japan	Case Report	1	19	Female	Poor
Ismail et al. [65]	Egypt	Case Report	1	48	Female	Fair
Jeon et al. [66]	Korea	Case Report	1	33	Female	Fair
Karasu et al. [67]	Turkey	Case Report	1	21	Female	Fair
Kasi et al. [68]	Korea	Case Report	1	22	Male	Poor
Kovach et al. [69]	United State	Case Report	1	68	Female	Fair
Kozlowski et al. [70]	South Africa	Case Report	1	30	Male	Poor
Kunavisarut et al. [71]	Thailand	Case Series	11	31	10 Females, 1 Males	Fair
Kusano et al. [72]	Japan	Case Series	7	66.28	7 Males	Fair
Kyung Cho et al. [73]	Korea	Case Report	1	44	Female	Fair
Lai et al. [74]	Hongkong	Case Report	1	26	Male	Fair
Lauer et al. [75]	Portland	Case Report	1	3	Male	Poor
Lemagne et al. [76]	Belgium	Case Report	1	46	Female	Fair
Li et al. [77]	China	Case Report	1	65	Male	Fair
Li et al. [78]	China	Case Report	1	16	Female	Fair
Liang et al. [79]	China	Case Report	1	16	Female	Poor
Lima et al. [80]	Chile	Case Series	2	51	2 Females	Fair
Lin et al. [81]	Taiwan	Case Report	1	50	Male	Fair
Lipper et al. [82]	USA	Case Report	1	16	Male	Fair
Liu et al. [83]	China	Case Report	1	21	Male	Poor
Lujan et al. [84]	USA	Case Report	1	71	Female	Poor
Madsen et al. [85]	Denmark	Case Series	6	20.33	3 Females, 3 Males	Fair
Mahmoud et al. [86]	Tunisia	Case Report	1	39	Male	Poor
Mäntyjärvi et al. [87]	Finland	Case Report	1	55	Male	Fair
Mariani et al. [88]	USA	Case Report	1	25	Male	Fair
Marr et al. [89]	N/A	Case Report	12	37.27	Male	Fair
Massa et al. [90]	Portugal	Case Report	1	53	Male	Fair
Mbekeani et al. [91]	USA	Case Report	1	30	Female	Poor
Meyer et al. [92]	Germany	Case Report	1	15	Male	Fair
Narendran et al. [93]	India	Case Report	1	64	Male	Fair
Nautiyal et al. [94]	USA	Case Report	1	53	Male	Fair
Nema et al. [95]	India	Case Report	1	23	Male	Poor
Okwuosa et al. [96]	USA	Case Report	1	27	Male	Fair
Olson et al. [97]	USA	Case Report	1	24	Female	Fair
Orzalesi et al. [98]	Kingdom of Sardinia	Case Series	2	50	2 Males	Poor
Park et al. [99]	UK	Case Report	1	71	Female	Fair
Rahman et al. [100]	USA	Case Report	1	58	Male	Good
Remky et al. [101]	USA	Case Report	1	28	Male	Poor
Saengsirinavin et al. [102]	Thailand	Case Report	1	35	Female	Poor
Sauer et al. [103]	France	Case Report	1	47	Male	Fair
Shah et al. [104]	USA	Case Report	1	63	Female	Poor
Shah & Gawas [105]	India	Case Report	1	30	Male	Fair
Sharma et al. [106]	India	Case Report	1	19	Male	Fair
Shroff et al. [107]	India	Case Report	1	32	Male	Fair
Shukla et al. [108]	India	Case Report	1	22	Female	Fair
Stassen et al. [109]	Scotland	Case Report	1	45	Male	Fair
Stoumbos et al. [110]	USA	Case Series	3	28.67	3 Females	Good
Subudhi et al. [111]	India	Case Report	1	28	Male	Fair
Tabatabaee et al. [112]	Iran	Case Report	1	54	Male	Fair
Tang et al. [113]	USA	Case Report	1	53	Female	Fair
Tariq et al. [114]	USA	Case Report	1	37	Female	Poor
Tatlipinar et al. [115]	Turkey	Case Report	1	22	Male	Fair
Thatcher et al. [116]	Canada	Case Report	1	37	Female	Fair
Vezzola et al. [117]	Italy	Case Report	1	21	Female	Fair
Viola et al. [118]	Italy	Case Report	1	5	Female	Fair
Wang et al. [11]	Taiwan	Case Report	1	17	Female	Fair
Wu et al. [12]	China	Case Series	7	21.1	7 Females	Good
Yalinbas et al. [119]	Turkey	Case Report	1	70	Female	Fair
Yan et al. [120]	China	Case Report	1	18	Female	Fair
Yang et al. [121]	Korea	Case Report	1	53	Female	Fair
Yao et al. [122]	Australia	Case Report	1	30	Female	Poor
Zaidi et al. [123]	USA	Case Report	1	21	Female	Fair
Zwolinska et al. [124]	Poland	Case Report	1	4.5	Male	Fair
**Total**			**168**	**34.62**	**Females: 49.11%**	

### Causes of Purtscher’s & Purtscher-like retinopathy

The causative co-morbidity of retinopathy for each patient in our review was recorded. About 80.37% had clear causative co-morbidity, with trauma being the leading cause. Head/face trauma and other site trauma were the leading causes of retinopathy in 22.62% and 17.26% of included subjects respectively. Other causes had little prevalence, except for SLE (13.1%) and acute pancreatitis (11.9%), which had a relatively higher occurrence. **Table 2** summarizes the prevalence of each causative co-morbidity in our review.

**Table 2:** Causes of Purtscher’s & Purtscher-like retinopathy.

Cause	No. (%)
Head and facial trauma	38 (22.62%)
Trauma to other body sites	29 (17.26%)
Systemic Lupus Erythematosus	22 (13.1%)
Hemolytic Uremic Syndrome	4 (2.38%)
Still disease	3 (1.8%)
Acute pancreatitis	20 (11.9%)
Cardiovascular events	9 (5.36%)
Malignancy	7 (4.17%)
COVID-19 infection	3 (1.8%)
**Total**	**135 (80.37%)**

### Diagnostic tests used for investigation

Patients were diagnosed by several methods, and reported diagnostic tests were aggregated in **Table 3**. Most patients were subjected to multiple diagnostic tests to assess different parts of the eye. In general, four tests were mostly acquired, namely fluorescence angiography (61.9%), fundus photography (55.95%), slit lamp (55.36%), and optical coherence tomography (OCT) (41.07%).

**Table 3:** Diagnostic tests used for investigation.

Diagnostic test used	No. (%)
Slit lamp	93 (55.36%)
Optical coherence tomography (OCT)	69 (41.07%)
Wide field image	10 (5.95%)
OCT angiography	29 (17.26%)
Electroretinography	4 (2.38%)
Fluorescence angiography	104 (61.9%)
Fundus photography	94 (55.95%)
Other*	20 (11.9%)

*Other tests included: Color vision tests, Indocyanine Green (ICG) angiography, & Ophthalmoscopy.

### Relationship between cause of retinopathy and visual prognosis

The relationship between causes of retinopathy and the prognosis for affected eyes has been assessed using Chi-square analysis. Prognosis for each eye was either explicitly stated or acquired from final best corrected visual acuity (BCVA) changes during the follow-up period. We defined good prognosis as any improvement in the final BCVA, while poor prognosis as worsening of final BCVA compared to presentation. Unfortunately, no significant correlation was observed between cause of retinopathy and the extent of effect. **Table 4** shows the relationship as described in cases.

**Table 4:** Relationship between cause of retinopathy and visual prognosis.

Cause of Retinopathy	Prognosis				
	Good	Poor	N/A*	Total	P-value
Head and facial trauma	19 (11.2%)	9 (5.3%)	10 (5.9%)	38 (22.62%)	0.871
Trauma to other body sites	15 (8.9%)	5 (3%)	9 (5.3%)	29 (17.26%)	0.793
Systemic Lupus Erythematosus	9 (5.3%)	5 (3%)	8 (4.7%)	22 (13.1%)	0.446
Hemolytic Uremic Syndrome	2 (1.2%)	1 (0.6%)	1 (0.6%)	4 (2.38%)	0.977
Still disease	2 (1.2%)	0 (0%)	1 (0.6%)	3 (1.8%)	0.671
Acute pancreatitis	11 (6.5%)	4 (2.4%)	5 (3%)	20 (11.9%)	0.967
Cardiovascular events	4 (2.4%)	3 (1.8%)	2 (1.2%)	9 (5.36%)	0.63
Malignancy	2 (1.2%)	2 (1.2%)	3 (1.8%)	7 (4.17%)	0.418
COVID-19 infection	3 (1.8%)	0 (0%)	0 (0%)	3 (1.8%)	0.253
**Total**	**89 (53%)**	**35 (20.8%)**	**44 (26.2%)**	**168 (100%)**	

*N/A: Not reported.

In patients, BCVA before and after treatment was used as an indicator for visual improvement/regression. Overall, patient showed great variety in terms of presentation BCVA, both OD and OS, however; after treatment, the trend was towards 0 logMAR BCVA, which corresponds to normal vision.

### Eye symptoms reported by patients

**Tables 5–8** summarize our findings regarding presenting symptoms, signs and finding in the retinas of patients. Regarding symptoms, they were centered around loss of vision, weather central or peripheral, blurring or sudden, without reports of pain. Of patients, 97 (57.7%) reported bilateral symptoms. The most reported symptom was OS centrally blurred vision at 34.32%, while OD central vision blurring was reported in approximately 18% of patients. Other symptoms were reported especially OD, including floaters, scotomas, ptosis, and color vision affection **(Table 5)**.

**Table 5:** Eye symptoms reported by patients.

Eye Symptoms	OD	OS
Bilateral: 97 (57.7%)		
Blurred or decreased Central vision	30 (17.75%)	58 (34.32%)
Blurred or decreased peripheral vision	26 (15.38%)	0 (0%)
Eye pain or discomfort	0 (0%)	0 (0%)
Sudden and painless vision loss	31 (18.34%)	35 (20.71%)
Visual field defects	4 (2.37%)	8 (4.73%)
Others*	44 (26.04%)	12 (7.1%)
**Total**	**77 (45.56%)**	**88 (52.07%)**

*Others include: Color vision drop, floaters, paracentral scotoma, Amsler grid changes, and ptosis.

**Table 6:** Eye signs reported in patients.

Eye Signs	OD	OS
Bilateral: 125 (74.4%)		
Retinal whitening	89 (52.9%)	84 (50%)
Cotton-wool spots	96 (56.8%)	98 (57.99%)
Relative afferent pupillary defect (RAPD)	15 (8.88%)	11 (6.51%)
Retinal hemorrhages	87 (51.48%)	81 (47.93%)
Swelling of the optic nerve.	34 (20.12%)	26 (15.38%)
Others*	31 (18.34%)	33 (19.53%)
**Total**	**122 (72.18%)**	**121 (71.6%)**

*Others include: Choroidal spots, disc margin blurring, intraretinal flame, patches of retinal pallor, retinal vascular engorgement, macular or optic disc edema, infarction of optic nerve fiber layer, microaneurysm, and hard exudates.

**Table 7:** Correlation between observed signs and visual prognosis.

Signs observed (per eye)	Poor Prognosis	Good Prognosis	P-value	Unknown prognosis*
Purtscher flecken	1 (0.3%)	61 (18.2%)	<0.01	109 (32.4%)
Cotton-wool spots	6 (1.8%)	73 (21.7%)	<0.01	115 (34.2%)
Retinal hemorrhages	4 (1.2%)	64 (19%)	0.012	100 (29.8%)
Swelling of the optic nerve	0 (0%)	16 (4.8%)	<0.01	44 (13.1%)
RAPD**	1 (0.3%)	14 (4.2%)	0.622	11 (3.3%)
Others***	6 (1.8%)	23 (6.8%)	<0.01	33 (9.8%)

*Unknown: Missing data, not included in the Chi-square statistic.

**RAPD: Relative Afferent Pupillary Defect.

***Others include: Choroidal spots, disc margin blurring, intraretinal flame, patches of retinal pallor, retinal vascular engorgement, retinal edema, infarction of nerve fiber, microaneurysm, and hard exudates.

**Table 8:** Types of retinal complications.

Retinal Complications	No. (%)
Macula edema	22 (13.02%)
Macular hole	1 (0.59%)
Retinal artery occlusion	3 (1.78%)
Retinal detachment	5 (2.96%)
Retinal tear or hole	0 (0%)
Retinal vein occlusion	9 (5.33%)
Vitreous hemorrhage	8 (4.73%)
Others*	16 (9.47%)
**Total**	**64 (37.87%)**

*Others include: Preretinal hemorrhage, acute macular neuroretinopathy (AMN), retinal atrophy, retinal fluid, splinter hemorrhage, venous leakage, and optic nerve atrophy.

### Eye signs reported in patients

**Table 6** summarizes signs observed in patients. Bilaterality was noted in about 75% of patients, which is significantly higher than reported symptoms. Cotton-wool spots were of highest prevalence, as they were reported OD in 56.8% of patients and OS in 58% of patients. Following, retinal whitening was seen in approximately 53% patients OD and 50% patients OS, then retinal hemorrhages was reported in around 50% of patients OD and OS. Several other signs were observed to a lesser extent.

### Correlation between observed signs and visual prognosis

In order to assess findings of prognostic visual value, Chi-square analysis was conducted. Interestingly Purtscher flecken (p<0.01), Cotton-wool spots (p<0.01), and optic nerve swelling (p<0.01) were associated with unknown or good visual prognosis **(Table 7**). No specific clinical finding was found to be associated with poor visual prognosis. However, data were insufficient to provide clear prognostic value of observed signs due to high missing data from the primary included studies.

### Types of retinal complications

Retinal complications were also recorded in patients. Most notably, macular edema was seen in 13% of patients. It is important to note that a wide range of retinal complications has been observed in patients. **Table 8** contains a summary for the prevalence of complications among patients.

### Treatment options utilized

Patients were managed according to their condition. In our review, treatments fell into six major categories, the most used of which was conservative management (60.7%). Systemic and topical corticosteroids in different doses were administered to 16.1% of included subjects. Combined anti-VEGF and laser therapy injections were used in 7.1%, and remaining modalities were acquired in small groups making 5% or less of patients (Table 9).

**Table 9:** Treatment options utilized.

Treatment Modality	No. (%)
Conservative observation	102 (60.7%)
Corticosteroids	27 (16.1%)
Anti-VEGF & Laser Therapy	12 (7.1%)
Anti-VEGF	8 (4.8%)
Laser therapy	8 (4.8%)
Intravitreal tissue plasminogen activator	1 (0.6%)
N/A	10 (6%)
**Total**	**168 00%)**

## Discussion

Purtscher’s and Purtscher-like retinopathy are sight threatening retinal conditions that manifest in relation to trauma or co-morbidity, respectively [2]. In this review, we attempt at a cross-comparison between published cases, in order to explore common factors in incidence, clinical picture, and prognosis. It is important to note that relatively low number cases are available in literature, as per our search, only 114 cases are available across all major medical research databases. Also, many published cases are of low quality. However, this review is the largest review discussing this rare entity.

The average age of included subjects is 34.62 years, which is consistent with similar cohorts in literature [5, 125]. However, our age group had a wide range, including patients as young as 2 years old, and as old as 75. Subjects were almost evenly distributed between males (50.89%) and females (49.11%). In this regard, literature suggests that males are subjected to head trauma or traumatic brain injuries at higher rates than females, reaching 40% more likelihood [126, 127]. On the contrary, acute pancreatitis, being the most reported co-morbidity with Purtscher-like retinopathy, is equally distributed between both sexes [128]. Of note, is the fact that epidemiological data for Purtscher’s and Purtshcer-like retinopathies is not sufficiently aggregated nor classified, making the identification of specific age/sex group as a risk group difficult.

In our review, only 80.37% of cases had clear related co-morbidity reported, which could hinder the full understanding of related causality. Nevertheless, of reported reasons, 67 (39.88%) patients reported a history of recent trauma, the majority of whom had head or facial trauma. This is reasonable, since the definition of Purtscher’s retinopathy includes the traumatic causality [2, 5]. As to Purtscher-like retinopathy, the most reported co-morbidity was SLE (13.1%) followed by acute pancreatitis (11.9%). Only one systematic review is available to compare these results, namely Miguel et al., and the findings in our study do not concede, as it reports acute pancreatitis to be the most reported co-morbidity [5]. However, our review has a much larger sample size, which gives more confidence in our findings. Both SLE and acute pancreatitis are associated with altered hemostasis, pre-disposing patients to retinal microangiopathy [46, 125]. But each of these diseases affects a different age group, as SLE is often manifested in younger individuals, i.e. second to third decade of life, while pancreatitis develops in older adults within their fifth to sixth decades [129, 130]. Thus, the connection between SLE and Purtscher’s retinopathy agrees with the findings regarding average age of patients affected. We conducted a chi-square analysis to assess the link between the predisposing co-morbidity and prognosis, with insignificant results due to poor available information. It was suggested in Miguel et al. study that male gender and etiological factors (acute pancreatitis and trauma) are of important prognostic value, but these findings are lacking in statistical power, in addition to being biased since most cases in the study are trauma or acute pancreatitis in the first place [5].

Information about the clinical picture in Purtscher’s and Purtscher-like retinopathies has been also recorded. Bilateral symptoms were reported in 97 (57.7%) patients, but upon examination, 75% of patients had bilateral findings. In the Miguel et al. study, bilaterality was reported in 14/68 (20.95%) cases [5]. Another study investigating Purtscher-like retinopathy in SLE patients reported bilaterality in 15/17 (88.2%) patients, however; this could be related to the nature of SLE, being a systemic multi-system disease [125].

Symptoms and signs did not have any tendency towards right or left eye and were evenly distributed. Patients complained mostly of centrally blurring of vision (OS: 34.32%, OD: 18%), however; these percentages suggest a high heterogenicity in symptoms presented.

Regarding signs, patients mostly had cotton-wool spots and retinal whitening. Retinal whitenings, known as Purtscher flecken are important features of Purtscher’s as the name implies [2]. Some literature considered that the presence of Purtscher flecken and cotton-wool spots along with etiological factors as a suitable diagnostic criterion for Purtscher’s and Purtscher-like retinopathies, which is questionable since Purtscher flecken were present in 50–53% of our patients only, and in 63.23% of patients in the Miguel et al. study [5, 125]. These findings manifest due to retinal artery occlusion by emboli of various origins and natures [2]. Retinal whitening manifests due to occlusion to the proximal retinal artery, while cotton-wool spots develop due to distal artery occlusion with small emboli [1]. Since distal arteries are of smaller diameter, smaller emboli are needed to exert effect, thus; cotton-wool spots are more likely to develop, which is what our data suggests. Retinal complications were specifically assessed, and were very diverse, making the most common complication macular edema which was seen in 13% of patients.

Overall, patients had a favorable prognosis (53%), which was also reported in an article by Schmidt and Otto on prognosis in Purtscher’s patients [131]. Although some complications are expected at a higher frequency as a result of the etiology of Purtshcer’s retinopathy, namely, trauma; No specific clinical finding was found to be associated with poor visual prognosis. Trauma-related complications as retinal detachment or vitreous hemorrhage were reported in a minority of patients (2.96% and 4.73%, respectively). This diversity of retinal complications can be attributed to the broad etiological base by which retinopathy develops, and our findings suggest the certain findings might have a significant prognostic value. Although about a third of reviewed articles did not provide clear prognostic description, we found that Purtscher flecken, cotton-wool spots, and optic nerve swelling might correlate to better improvement and favorable prognosis (p<0.01). However, the findings Schmidt and Otto suggest otherwise, as their work finds Purtscher flecken to correspond with worse prognosis. In addition, depending on case reports and series to provide strong evidence of the prognostic value couldn’t be conclusive.

Treatment of Purtshcer’s and Purtscher-like retinopathy are rather limited, especially with its ambiguous pathology. Generally speaking, each case should be individually assessed and managed, nevertheless; certain clinical features could help guide physicians. Since most patients were managed conservatively (60.7%), the primary clinical presentation is often indicative of no further complication, hence; no further intervention would be needed. In cases where complications develop, each complication calls for a suitable treatment modality. In our cohort, according to presentation and complications, modalities as corticosteroid-based management (16.1%), and combined anti-VEGF & laser therapy (7.1%) were used. A systematic review conducted by Xia et al. investigated the efficacy of Purtscher’s treatments on 139 eyes of 88 patients. In their study, glucocorticoids were used the most, in 63.29% of eyes, and conservative observation was used in 43.17% [132]. This suggests that management of Purtscher’s retinopathy patients can show great variety. In the Xia et al. study, patients showed improved vision across all groups, and no significant differences between treatment modalities was found. Moreover; similar to our observation, the quality of available evidence in this regard is rather poor, and no conclusive statements can be made [132]. A direct link between etiology-complication-treatment cannot be drawn from available information, moreover; treatment of Purtscher’s and Purtscher-like retinopathies often tend to the underlying systemic disease (e.g. SLE or Pancreatitis), making a specific management criterion tailored to Purtshcer’s or Purtscher-like retinopathy difficult to develop. Future research in this regard could assist in developing treatment guidelines for Purtscher’s and Purtscher-like retinopathies.

Limitations to this study can be summarized in the scarcity of available data, as well as the low quality of most cases reported. Future research in this topic should consider this limitation, as by overcoming them, a robust diagnostic criterion for Purtscher’s and Purtscher-like retinopathies can be developed. In addition, the nature of case reports and series may introduce bias and limit the generalizability of our findings raising questionable associations and prognostic values. Moreover, future research should pay attention to establishing a conventional treatment modality for these conditions, and to recognizing prognostic factors in order to prevent visual deterioration and loss.

## Conclusion

Purtscher’s and Purtscher-like retinopathies are rare sight-threatening retinopathies that develop most commonly following trauma or other systemic diseases as SLE and acute pancreatitis. Little data is available regarding these conditions, and available data is of low quality. Patients develop bilateral disease in approximately 50% of cases, and several retinal findings are observed, with no specific tendency. Most observed signs are cotton-wool spots in around 55% of patients and Purtscher flecken in 51% of patients. Patients spontaneously recovered, although data is not conclusive. No clear prognostic value of etiological factors is identified, and further research is required in this regard.

## Supporting information

S1 Checklist PRISMA checklist. (PDF)
